# Stenosis of the Trachea

**Published:** 1909-03-27

**Authors:** 


### Laryngology and Rhinology.

### STENOSIS OF THE TRACHEA.

Ætiology.

The causation of laryngeal stenosis was described in a previous article, and we will now discuss the causes of stenosis of the trachea, which may be conveniently divided into extra-tracheal and intra-tracheal.

Owing to its anatomical relations, compression of the trachea by adjacent structures not infrequently produces narrowing of its lumen; as a result of continued pressure the cartilaginous rings become gradually softened and are then more readily bent inwards. Tumours of the thyroid gland, aneurysms, enlarged lymphatic glands within the thorax, and mediastinal tumours are among the relatively common causes of this form of stenosis; hypertrophy of the thymus gland in children may also be mentioned, and, as rare causes, extreme pleural and pericardial effusion and dilatation of the left auricle.

The trachea is much softer and more compressible in young children than in adults, and is therefore more readily narrowed by such causes as these. Goitre is perhaps the commonest cause of compression; any form of goitre, innocent as well as malignant, may produce this stenosis, provided only that the enlargement is fairly rapid. The compression may be well seen with the laryngeal mirror and, when both lobes of the gland are enlarged, results in the so-called " scabbard-shaped " stenosis; when only one lobe is affected the antero-lateral aspect of the tracheal wall may be seen to be bulged inwards.

Aneurysm and Enlarged Thymus.

An aneurysm compresses the trachea lower down, near the bifurcation, and occasionally the resulting prominence, or even pulsation, may be seen with the laryngoscope, especially if the observer sits while the patient stands above him with the head bent; this position, originally suggested by Killian for inspection of the posterior wall of the larynx, generally gives the best view of the lower end of the trachea. An aneurysm may open into the trachea, but usually does so very gradually; a slight leaking of blood is the first symptom, and sometimes continues intermittently for many days before the final rupture.

There has been much difference of opinion, and not a little lively discussion, concerning the part played by enlargement of the thymus gland in the production of dyspnœa in children. Hypertrophy of the thymus has doubtless, without sufficient reason, been ascribed as the cause of infantile dyspnœa due in reality to spasmodic laryngitis, laryngismus stridulus, or that peculiar malformation of the larynx which produces congenital laryngeal stridor, and a distinct affection has even been described under the name of "thymic asthma." Although it is probable that most of these cases are not due to the thymus, there is little doubt that exceptional hypertrophy of this gland may compress the trachea. The subject is closely connected with that of sudden death due to the condition known as the " status lymphaticus," in which the thymus and all the lymphatic structures are enlarged.

Intra-tracheal Causes.

Foreign bodies and diphtheritic membrane; traumata, such as cut-throat, fractures of the tracheal rings, and injuries resulting from the operation of tracheotomy; gummata; neoplasms are rare, but malignant growths of the thyroid gland occasionally penetrate into the lumen of the trachea; and, finally, cicatricial contraction following syphilitic ulceration or after injuries or tracheotomy. Cicatricial contraction is the only form of stenosis which is seen at all frequently, and is generally due to syphilis; one may see a sharp white border of scar-tissue surrounding a round or oval hole, an appearance totally different from the rounded projection caused by external compression. Stenosis after tracheotomy, causing difficulty in removing the tube, may be set up by depression of the anterior wall of the trachea by the tube pushed through too small an opening, by kinking of the posterior wall, by granulations sprouting into the lumen, or by cicatricial contraction following ulceration. Difficulty in dispensing with the tube is, however, very frequently due, not to any organic stenosis, but to spasm caused by nervousness. When organic stenosis is present it is generally found that the attempted tracheotomy has been performed too high, and that the cricoid cartilage has been divided, or even that the tube has been inserted through the thyroid cartilage.

Symptoms.

Naturally the principal symptom of stenosis is dyspnœa, which in severe cases is continuous, but in early or slighter degrees occurs only on exertion or in exacerbations due to spasm; the latter is an important factor in strictures of any of the canals in the body. There is marked retraction of the intercostal spaces and of the epigastric and supra-clavicular regions, which helps at a glance to distinguish the dyspnœa of stenosis from that due to deficient aeration, as in cardiac disease. Patients with laryngeal stenosis sit propped up with the head thrown back. There is marked stridor during inspiration, while expiration is comparatively easy. An important sign is the drawing downwards of the larynx with each inspiration; the voice is nearly always affected except in cases of abductor paralysis.

On the other hand, in tracheal stenosis the patient generally leans forward to avoid stretching the trachea, the stridor is as marked in expiration as inspiration, the larynx is not sucked downwards, and the voice, though weak, is not hoarse. The dyspnœa of asthma, with its short inspiration and characteristic, prolonged, wheezy expiration, is quite distinctive.

